# Residual Effects of 50-Year-Term Different Rotations and Continued Bare Fallow on Soil CO_2_ Emission, Earthworms, and Fertility for Wheat Crops

**DOI:** 10.3390/plants11101279

**Published:** 2022-05-10

**Authors:** Lina Skinulienė, Aušra Marcinkevičienė, Lina Marija Butkevičienė, Vaida Steponavičienė, Ernestas Petrauskas, Vaclovas Bogužas

**Affiliations:** Agroecosystems and Soil Sciences Department, Vytautas Magnus University, K. Donelaičio Str. 58, 44248 Kaunas, Lithuania; ausra.marcinkeviciene@vdu.lt (A.M.); lina.butkeviciene@vdu.lt (L.M.B.); vaida.steponaviciene@vdu.lt (V.S.); ernestas@agronom.lt (E.P.); vaclovas.boguzas@vdu.lt (V.B.)

**Keywords:** soil physicochemical and biological properties, soil CO_2_ emission, crop rotation, pre-crop, continuous bare fallow, perennial grasses, wheat yield

## Abstract

In this study, our investigated hypothesis was that different pre-crops would have different effects on earthworm activity and soil CO_2_ emissions. We also hypothesized that a pre-crop clover–timothy mixture would perform best in terms of increasing the share of organic carbon in soil and, in this way, contribute to improving the sustainability of agroecosystems. The aim of this study was to explore the residual effects of using a 50-year-term of three different crop rotations and a continuous bare fallow period on soil CO_2_ emissions by investigating the soil earthworm populations, soil agrochemical properties, and winter wheat yields. A field experiment was carried out from 2016 to 2017 at Vytautas Magnus University in Lithuania (54°53′ N, 23°50′ E). The experiment was conducted in crop stands of winter wheat cv. ‘Skagen’, which were sown in three crop rotations with different pre-crops and a continuous bare fallow period. The pre-crop used for winter wheat in the cereal crop rotation (CE) was a vetch and oat mixture for green forage, LEG-CER; the pre-crop used for winter wheat in the field with row crops (FWR) crop rotation was black fallow, FAL-CER; the pre-crop used for winter wheat in the Norfolk (NOR) crop rotation was a clover–timothy mixture, GRS-CER; and finally, continuous bare fallow, FAL-CONTROL, was used as well. The highest soil CO_2_ emission intensity was determined after the pre-crops that left a large amount of plant residues (clover and timothy mixture) in the soil. Plant residues remaining after the pre-crop had the greatest effect on the number of earthworms in the soil after the harvesting of winter wheat. Winter wheat had the best yield when grown in grass and legume sequences. Crop rotation sequences that included perennial grasses accumulated higher contents of total nitrogen and organic carbon. The best values for the productivity indicators of wheat were obtained when it was grown after a fallow crop fertilized with cattle manure. An appropriate crop rotation that promotes the steady long-term contribution of organic matter and increases the content of organic carbon in the soil will have a positive effect on the agrochemical, biological, and physical properties of soil and agroecosystem sustainability; moreover, these effects cannot be achieved by technological means alone.

## 1. Introduction

Soil is one of the most important means of carbon storage in the biosphere. The way in which soil is utilized has a direct impact on soil CO_2_ emissions to the atmosphere [1]. Choosing the appropriate soil utilization has the potential to mitigate both the current and future impacts of climate change [2,3].

Earthworms, which are known as ecosystem engineers, play an indispensable role in shaping soil structure and supplying soil with nutrients that are accessible to plants [4]. By promoting the decomposition of organic matter, earthworms accelerate nitrogen mineralization, improve nutrient availability [5], increase the activity of soil microbes, and thus enhance plant growth [6]. Because of these effects, earthworms have a major impact on soil properties [7]. Laossi et al. [7] found that earthworms are involved in the formation of organic–mineral colloids, regulating the water and air balance in the soil while reducing the prevalence of plant diseases [8]. Lemtiri et al. [9] confirmed this in their research by describing how earthworms play an active role in treating organic residues by accelerating humification. Earthworm abundance has been found to be higher in crop rotations that incorporate high levels of organic matter into the soil [10].

The quantity and quality of crop residues remaining in the soil have an impact on soil carbon dioxide emissions, level of SOC (soil organic carbon), and the amount of easily mineralized carbon it contains, all of which depend on the crop rotation used in the agricultural system [11,12]. Some studies have observed that growing crops, using large amounts of mineral fertilizers, and introducing little organic matter into the soil can cause a significant decrease in organic matter, the consequences of which are reflected in cereal productivity [13].

One of the most pressing challenges at present is adapting to the impacts of climate change. This is a key issue for both the planning and implementation of sustainable agriculture [14], and is particularly relevant for winter crop productivity [15]. One of the strategies used in soil management is the selection of an appropriate crop sequence scheme, which will help to adapt to nutrient needs, maintain soil health, and simultaneously reduce the incidence of pests and diseases. All this should be completed in order to achieve the ultimate goal of maintaining a sustainable and at the same time high level of production [16]. Meeting the need for fodder and fiber production worldwide requires soil to remain consistently productive [17]. Crop rotation, in which soil-depleting crops are replaced by soil-improving crops, is one of the solutions used to preserve soil health. Wheat (*Triticum aestivum* L.) is one of the most important crops in terms of its nutritional value and other useful properties for human health. Wheat is grown worldwide under different climatic conditions and ranks second in terms of the level of production after maize (*Zea mays* L.) globally [18].

We hypothesized that the use of different pre-crops would increase earthworm activity and decrease soil CO_2_ emissions, while the use of a pre-crop clover–timothy mixture would perform best in terms of increasing the share of organic carbon in soil and, in this way, contributing to the sustainability of agroecosystems.

The aim of this study was to explore residual effects of a 50-year-term of three different crop rotations and a continuous bare fallow period on the soil CO_2_ emissions, earthworm population, agrochemical properties, and winter wheat yield.

## 2. Results

### 2.1. Soil CO_2_ Emissions, Temperature, and Moisture

In this study, the effect of the use of long-term crop rotation combinations and fallow periods on soil CO_2_ emissions was estimated three times: at the beginning of the study period, in the middle of the experiment, and the end. Three different crop rotations with different pre-crops were investigated: the pre-crop used for winter wheat in the cereal crop rotation (CE) was a vetch and oat mixture for green forage, LEG-CER; the pre-crop used for winter wheat in the field with row crops (FWR) crop rotation was black fallow, FAL-CER; the pre-crop used for winter wheat in the Norfolk (NOR) crop rotation was clover–timothy mixture GRS-CER; and continuous bare fallow, FAL-CONTROL, was used as well. The BBCH scale (Biologische Bundesanstalt, Bundessortenamt and CHemical industry) was used to identify the phenological development stages of plants.

**Figure 1:**
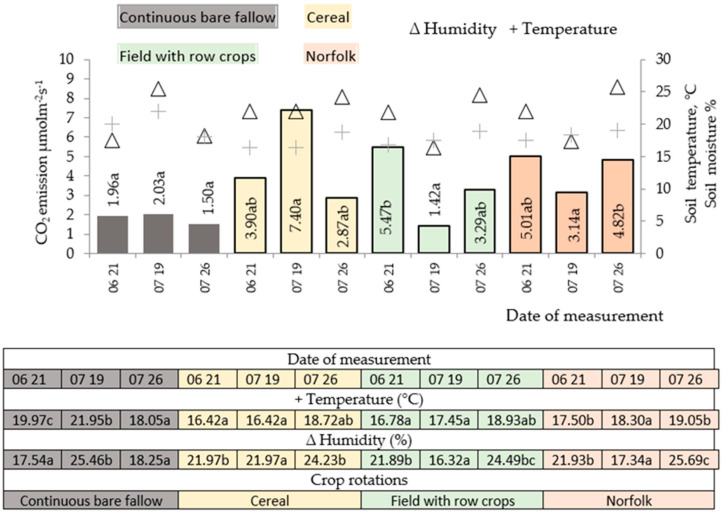
Soil moisture, temperature, and CO_2_ emissions for the 2016 winter wheat crop. Note: a–c indicate significant differences between the treatments (*p* ≤ 0.05). FAL-CONTROL, continuous bare fallow; LEG-CER (CE), winter wheat pre-crop was a vetch and oat mixture for green forage while the crop rotation was cereal; FAL-CER (FWR), winter wheat pre-crop was black fallow while the crop rotation was field with row crops; GRS-CER (NOR), winter wheat pre-crop was a clover–timothy mixture while the crop rotation was Norfolk.

In 2016, in winter wheat crops, the soil CO_2_ emissions during the first measurement period (BBCH 68–70) were the most intense in the field crop rotation with cumulative crops. In this field, black fallow was maintained before winter wheat and fertilized with cattle manure before winter sowing took place. However, significantly lower soil CO_2_ emissions were found in the control fallow (Figure 1). No significant differences in soil CO_2_ emissions were found between the crop rotations of winter wheat. 

The most intense soil respiration occurred after the wheat started maturing (BBCH 78–83) in the cereal crop rotation in which the pre-crop used for winter wheat was a mixture of vetch and oats fertilized with cattle manure, where the value obtained was on average 3.8 times higher compared to that obtained for the other crop rotations. At this stage, the soil CO_2_ emissions were the lowest in the field with cumulative crops. The lowest soil CO_2_ emissions in spring were found for the Norfolk crop rotation; these values did not differ significantly from those of the control fallow.

During the hard-ripening stage of winter wheat (BBCH 87–89), the soil CO_2_ emissions were similar in all crop rotations. The most intense and stable soil CO_2_ emissions throughout the entire vegetation period were found for the Norfolk crop rotation, when winter wheat was preceded by perennial grasses that provided abundant organic residues.

It can be assumed that in black fallow, organic fertilizers particularly promote mineralization at favorable times—i.e., in the spring. However, this process slowed due to the lack of moisture during the vegetation period. In crop rotations in which the pre-crop used for winter wheat was beans, the mineralization process was activated in the middle of the vegetation period and remained stable throughout the vegetation period after perennial grasses.

No significant differences were found between the crop rotations throughout the winter wheat growing season. During the third measurement (winter wheat hard maturity stage, BBCH 87–89), the most intensive and stable soil CO_2_ emissions were identified in the crop rotation where winter wheat was preceded by a mixture of perennial grasses, leaving abundant organic residues.

**Figure 2:**
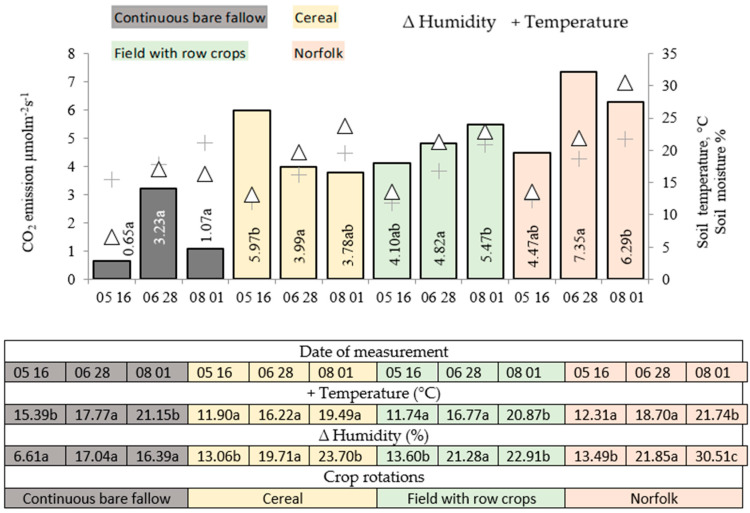
Soil moisture, temperature, and CO_2_ emissions in the 2017 winter wheat crop. Note: a–c indicate significant differences between the treatments (*p* ≤ 0.05). FAL-CONTROL, continuous bare fallow; LEG-CER (CE), winter wheat pre-crop was a vetch and oat mixture for green forage while the crop rotation was cereal; FAL-CER (FWR), winter wheat pre-crop was black fallow while the crop rotation was field with row crops; GRS-CER (NOR), winter wheat pre-crop was a clover–timothy mixture while the crop rotation was Norfolk.

In 2017, in the cereal crop rotation, intensive organic matter decomposition after the application of a vetch and oat mixture occurred at the beginning of the winter wheat growing season (Figure 2). In the field crop rotation with row crops, soil CO_2_ emissions were found to increase with each measurement. In the latter crop rotation, winter wheat was sown in fallow fertilized with cattle manure.

In the crop rotations for 2017, the soil CO_2_ emissions were 7.4 times more intense compared to those of the control fallow (Figure 2). In the control fallow, the intensity of emissions increased only in the middle of the vegetation period. The degradation of organic matter was significantly more intense in the crop rotation after the mixture of vetch and oats was applied at the beginning of wheat vegetation (BBCH 37–39) and decreased by an average of 34.0% by the end of the vegetation period (BBCH 87–89).

Soil CO_2_ emissions varied differently in the field with cumulative crops and in the Norfolk crop rotation. At the beginning of the vegetation period (BBCH 37–39), the emission intensity was similar to that of the previous year, and by the end of the vegetation period (BBCH 87–89) the crop rotation of the field with cumulative crops had increased on average by 13.2%. Soil CO_2_ emissions from the Norfolk crop rotation were particularly high in the middle of vegetation period (BBCH 78–83) and persisted until the end of the period (BBCH 87–89) (1.5 times higher than at the first measurement).

Mineralization processes in the soil are influenced by the amount of organic matter present and the moisture regime. The year 2017 was wetter than 2016, especially at the end of the vegetation period. This was a determining factor in the level of soil CO_2_ emissions in 2017. Another influencing factor was the abundance of vegetative residues and the duration of their decomposition: in the Norfolk crop rotation, the residues of perennial grasses decomposed more slowly than those of the mixture of vetch and oats with cattle manure, so the soil CO_2_ emissions were stable until the end of the vegetation period and quite intense in wet years. Only cattle manure was applied in the black fallow before wheat sowing (FWR), with the soil CO_2_ emissions increasing in the wet years until the end of the vegetation period. However, in the dry years, the emissions were equal to those of the control fallow. To reduce the intensity of soil CO_2_ emissions, it is better to fertilize the pre-crop with an organic fertilizer. The results of this study showed that in the cereal crop rotation, the fertilization of pre-crops with cattle manure reduced the intensity of soil CO_2_ emissions at the end of the wheat vegetation period both in the drier year 2016 and the wetter year 2017.

### 2.2. Number and Mass of Earthworms in the Soil

**Figure 3:**
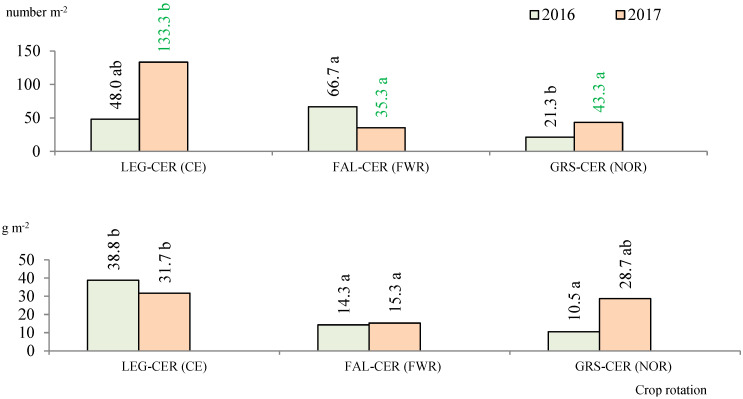
Number and mass of earthworms in the winter wheat crops in 2016 and 2017. Note: a,b indicate significant differences between the treatments (*p* ≤ 0.05). FAL-CONTROL, continuous bare fallow; LEG-CER (CE), winter wheat pre-crop was a vetch and oat mixture for green forage while the crop rotation was cereal; FAL-CER (FWR), winter wheat pre-crop was black fallow while the crop rotation was field with row crops; GRS-CER (NOR), winter wheat pre-crop was a clover–timothy mixture while the crop rotation was Norfolk.

In 2016, after winter wheat harvesting had taken place, the greatest number of earthworms was found in the stubble of the crop rotation with row crops. In the stubble of the cereal crop rotation, the number of earthworms found was lower; however, the difference was insignificant. Meanwhile, in the Norfolk crop rotation the earthworm number was 3.1 times lower (Figure 3), representing a significant decrease. A significantly higher (from 2.7 to 3.7 times) earthworm mass was recorded in the cereal crop rotation where a vetch and oat mixture had preceded winter wheat. In the field crop rotation with row crops, winter wheat is fertilized with organic fertilizers, and in the cereal crop rotation the pre-crop is fertilized. In the Norfolk crop rotation, winter wheat is also fertilized with cattle manure and sown after the perennial grasses of the first harvest year, but in 2016 a decrease in the earthworm number and mass was observed. 

In 2017, the number of earthworms in the stubble of the winter wheat in the cereal crop rotation was significantly (3.1 times) higher than that in the Norfolk crop rotation and 3.8 times higher than that in the field crop rotation with row crops (Figure 3). Compared to the cereal crop rotation, a significantly lower (3.8 times) number of earthworms were found in the field crop rotation with row crops, in which winter wheat was sown after bare fallow, but no significant differences were observed in earthworm mass. That year, the earthworm number and mass in the Norfolk crop rotation were also higher than those in the field crop rotation with row crops but lower than those in the cereal crop rotation. No earthworms were found in the continuous bare fallow due to the low organic matter content. The amount of rainfall in July was 21.3% lower than the long-term average, and in August it was 61.6% lower. 

### 2.3. Soil Agrochemical Properties

Winter wheat was cultivated in the cereal, field with row, and Norfolk crop rotations (Table 1). In the cereal crop rotation, the winter wheat pre-crop used for green fodder was a mixture of vetch and oats. In the field with the row crop rotation, the pre-crop used for winter wheat was bare fallow, while the pre-crop used in the Norfolk crop rotation was multiannual leys. The highest content of organic carbon (15.2 g/kg) was found for the winter wheat crop in the Norfolk crop rotation. In the cereal crop rotation, the content of organic carbon was 1.1 to 1.2 times lower than that in the other crop rotations investigated.

The largest amounts of available phosphorus recorded in 2016 in the winter wheat crops were found in crop rotations with cereals (388.33 mg/kg) and raw crops (371.93 mg kg^−1^). The winter wheat pre-crop used in the cereal crop rotation was a mixture of vetch and oats, while in the field with row crops one year of bare fallow was used (Table 2).

The cultivation of *Poaceae* plants (grasses), and, in particular, their reseeding, reduces the content of organic carbon in the soil. In the cereal crop rotation, grasses made up 75% of the crop structure, while in the field with row crops they made up 50%. Thus, it needs to be explained why these crop rotations had, on average, a 19.3% lower carbon content compared to the Norfolk crop rotation (Table 1). Although wheat is not reseeded, the presence of a large proportion of it in the crop structure reduces the organic carbon stock in the soil. Norfolk is a four-course crop rotation where the crop structure is 50% cereals, but bare fallow is not applied as in the field with row crops. In continuous bare fallow, the carbon content was found to be significantly lower compared to the crop rotations by an average of 39.6%.

The level of total nitrogen was the highest in the Norfolk crop rotation. The use of a pre-crop of perennial grasses increased the reserves of total nitrogen in the soil for the wheat crop significantly (by 27.1%) compared to the pre-crop of vetch and oat mixture used in the cereal crop rotation, as well as increasing it by 20.3% compared to wheat sown in bare fallow. Fertilization with organic fertilizers also did not increase the total nitrogen content. The storage of continuous bare fallow significantly reduced the reserves of nitrogen in the soil compared to the crop rotations by 38.6% on average.

In the winter wheat crops, the phosphorus and potassium levels differed in different crop rotations after the use of different pre-crops (Table 1). The highest concentration of phosphorus (348.2 mg/kg) was found in the Norfolk crop rotation, and the concentration of potassium was 431.6 mg/kg. A higher level of potassium was found in the cereal crop rotation, but this difference was insignificant, at only 5.0%. In this crop rotation, the level of phosphorus was lower compared to that of cereals, but again only insignificantly. In the soil of the field with row wheat crops, the amount of phosphorus was found to be 11.4% lower on average compared to that of the Norfolk and cereal crop rotations, but the difference was insignificant. The decrease in the content of potassium in the aforementioned crop rotation was only significant compared to that of the cereal crop rotation—15.4%. The storage of continuous bare fallow significantly reduced the content of phosphorus (1.6 times) and that of potassium (2.0 times) in the soil compared to the contents of these elements in the crop rotations.

In the control fallow, all the nutrient stocks showed decreases. Leaving black fallow before sowing wheat even for one year in the crop rotations of eight fields reduces the stock of macronutrients and carbon in the soil compared to that available when a pre-crop is grown. The use of the short Norfolk crop rotation when producing perennial grasses and fertilizing with cattle manure significantly increases the soil nutrient stocks compared to those seen in other crop rotations. Nevertheless, this increases the soil CO_2_ emissions as well.

### 2.4. Cereal Yield Productivity 

In the collection of crop rotations, winter wheat was grown in legume (after a vetch–oat mixture), fallow (after one year of bare fallow), and grass (after perennial grasses of the second harvest year) sequences. 

In 2016, no significant differences in wheat yield were found, but a positive tendency was established when wheat was sown after a vetch–oat mixture that was fertilized with cattle manure.

**Figure 4:**
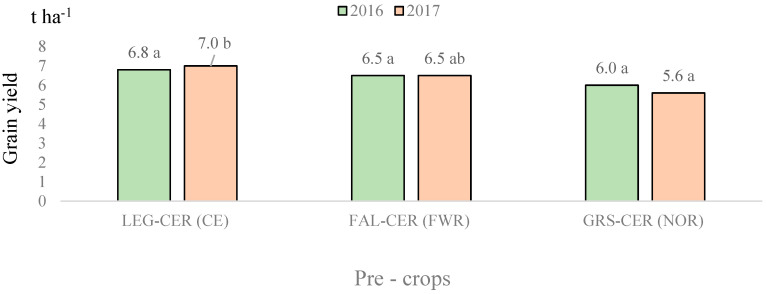
Winter wheat productivity after the use of various pre-crops in different crop rotations in 2016 and 2017. Note: a,b indicate significant differences between the treatments (*p* ≤ 0.05). FAL-CONTROL, continuous bare fallow; LEG-CER (CE), winter wheat pre-crop was a vetch and oat mixture for green forage while the crop rotation was cereal; FAL-CER (FWR), winter wheat pre-crop was black fallow while the crop rotation was field with row crops; GRS-CER (NOR), winter wheat pre-crop was a clover–timothy mixture while the crop rotation was Norfolk.

In 2017, the trends seen in the two previous years repeated: winter wheat showed the best yield in the legume sequence when grown in cropped fallow after cattle manure was applied to a vetch–oat mixture. The productivity of wheat after perennial grasses were grown for one year was the lowest and significantly (19.8%) differed from that of wheat grown in a cropped fallow (Figure 4). The productivity of wheat grown in bare fallow did not differ significantly from that of wheat grown in both the legume and grass sequences. 

No organic fertilizers were applied in the green manure and fodder crop rotations, while in other crop rotations one field was treated with organic fertilizers. Cattle manure was found to influence the cereal productivity in different crop rotations; however, fertilization with manure alone is insufficient to ensure a favorable plant nutrition regime. 

**Figure 5:**
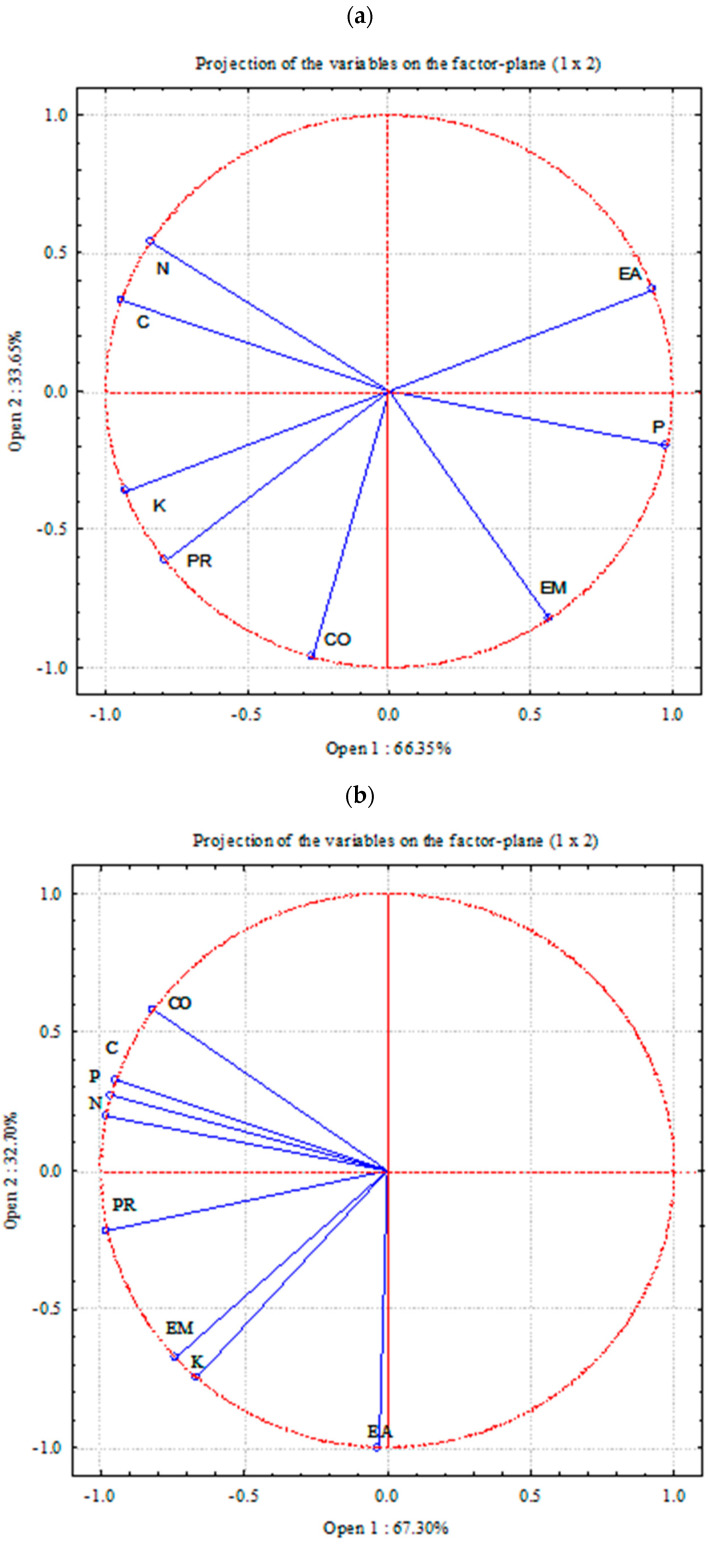
Relationships between different parameters depending on the crop rotations used in 2016 (Figure (**a**)) and 2017 (Figure (**b**)). Note: N, total nitrogen; C, organic carbon; K, available potassium; P, available phosphorus; CO, soil CO_2_ emission; EA, amount of earthworms; EM, mass of earthworms; PR, productivity.

Our analysis showed the existence of different relations between individual parameters depending on the crop rotations used. In 2016, the variables formed three groups (Figure 5). The first group of correlated parameters was the content of organic carbon and total nitrogen. The second group was the content of available potassium, winter wheat productivity, and, to a lesser extent, soil CO_2_ emissions. The third group of correlated parameters was the content of available phosphorus and the mass of earthworms.

In 2017, the mass of earthworms was found to depend on the content of available potassium. Winter wheat productivity correlated most with the content of total nitrogen, available phosphorus, and organic carbon. This analysis shows which indicators have correlations and which are completely unrelated. A principal components analysis was used to create new artificial variables (principal components) based on the variables that we analyzed.

## 3. Discussion

Carbon dioxide is a basic greenhouse gas [19]. Agriculture directly contributes approximately 12% of the annual anthropogenic greenhouse gas (GHG) emissions [20], 39% of which comes directly from soils [21]. Soil CO_2_ emissions is a good indicator of soil biological activity [22]. The first measurement of this parameter (BBCH 37–39) showed that the most intensive period of soil CO_2_ emissions occurred in 2016 in the field crop rotation with row crops where winter wheat was preceded by bare fallow fertilized with cattle manure. Feizienė et al. [23] suggest that in farming systems which incorporate more organic residues in soil, the vital functions are more intensive. They claim that soils that are not fertilized with synthetic fertilizers and not exposed to pesticides retain their naturally viable microbiological environment. In the spring of 2017, the highest soil CO_2_ emissions were recorded in the early stage of cereal growth (BBCH 37-39) in the cereal crop rotation, in which the pre-crop used for winter wheat was fertilized with a mixture of vetch and oat. The mixture of organic fertilizers and grasses added nitrogen to the soil, promoting the decomposition of crop residues while increasing the soil CO_2_ emissions. Some studies have shown that when using a combination of crop rotations, for straw decomposition it is necessary to incorporate additional nitrogen fertilizers in order to increase soil biological activity and straw mineralization [24,25,26]. Lee et al. [27] found that soil CO_2_ emissions depend on the crop species used and the growth stage. In 2017, in the cereal crop rotation, intensive organic matter decomposition occurred at the beginning of winter wheat growing season after the application of the vetch and oat mixture. The decomposition of pre-crop green manure and mineral nitrogen release in the soil are affected by soil moisture and temperature along with the chemical composition of the plants incorporated [28]. It was found that the intensity of soil CO_2_ emissions decreased at the end of the cereal vegetation period in 2016 in all crop rotations, but an increase in soil moisture content was also observed during that period. A similar trend was identified in the cereal and Norfolk crop rotations in 2017. In the black fallow, the intensity of soil CO_2_ emissions decreased due to nutrient deficiencies rather than the humidity or temperature. Agronomic factors influencing soil CO_2_ emissions into the atmosphere include the method of soil cultivation used, the introduction of organic matter, and mineral fertilization [29]. After sowing winter wheat in the bare fallow fertilized with cattle manure, soil CO_2_ emissions increased throughout the growing season. In crop rotations where the pre-crop used was legumes, the intensity of the soil CO_2_ emissions depended on the type of legumes used and the fertilization method applied. In the crop rotation that followed the application of the mixture of vetch and oat, the emissions were highest in the middle of the vegetation period (BBCH 68–70) in 2016 and in the spring (BBCH 37–39) of 2017. In the Norfolk crop rotation, the pre-crop used for wheat was perennial grasses, and the CO_2_ emissions remained stable throughout the growing season. In the continuous bare fallow without the use of fertilizers and where no plants were grown, the soil CO_2_ emissions were the lowest and their storage was not economically beneficial. Stabilizing greenhouse gas emissions from croplands as agricultural demand grows is a critical component of climate change mitigation strategies [29,30,31]. Moreover, several researchers have reported that earthworms play an important role in the emissions of GHGs from soil, since they can significantly influence the physical and chemical properties of habitats and thereby affect the production and emissions of GHGs [32,33]. After the cereal harvest in 2016, the number of earthworms per square meter was the highest in the field with row crops, but their mass was, on average, as much as 2.7 times lower than that of earthworms found in the cereal and Norfolk crop rotations. Fertilization with organic fertilizers promotes the reproduction of earthworms, but constant tillage reduces their population and mass, meaning that the earthworms are small; in our experiment, their number decreased significantly in 2017. In crop rotations where the pre-crop leaves some organic matter and less tillage operations are applied, the number of earthworms will be more stable. Under favorable conditions, the number of earthworms and their mass will increase. The use of potatoes in the Norfolk crop rotation did not increase the number of earthworms, but the plant residues left by perennial grasses did increase their mass. Intensive tillage may have had an effect: the potatoes present in the crop structure were earthed 4–5 times per growing season and the clover fields were prepared for winter cereals after the first harvest of herbage was brought in during the middle of summer. The number of earthworms present showed that there were sufficient nutrients for them to feed and procreate. Earthworms protect C in aggregates even as they increase soil respiration [34,35] and boost both above- and belowground plant inputs to soil [36].

Carbon concentrations depend on the origin of the soil, its horizon (layer), the vegetation present, and the fertilization methods applied [37,38]. The cultivation of grasses, and, in particular, their reseeding, reduces the content of organic carbon in the soil. The use of a pre-crop of perennial grasses in a short Norfolk crop rotation increased the organic carbon content in the soil and reduced the level of nitrogen significantly more so than the use of organic fertilizers before sowing wheat in bare fallow or in a mixture of vetch and oat fertilized with manure. It is necessary to explain why the soil CO_2_ emissions in the Norfolk crop rotation were similar throughout the growing season. These emissions could be caused by the soil organic matter in the agroecosystem mainly being organic matter produced by plant photosynthesis and autotrophic soil bacteria, as well as organic residues of animal origin and organic fertilizers [39]. Some studies have found a close relationship between the content of soluble C and soil microbiological activity, and between the content of water-soluble C and clay particles in the soil, as clay minerals actively absorb dissolved organic compounds when moving down the profile [40,41,42]. The results of the experiment showed that the way the soil is used has a significant effect on the amount of nutrients in a winter wheat crop. In the soil of the continuous bare fallow field, all nutrient parameters were significantly lower compared to those in the soil used for the wheat crop rotation. In the field with row crops, the use of a bare fallow period every 8 years, despite the field being fertilized with manure, significantly reduced the contents of organic carbon and total nitrogen, and caused less phosphorus and potassium to be found. In the crop structure of the cereal crop rotation, where 75% grasses were used and the soil was fertilized with organic fertilizers every 4 years, the contents of carbon and nitrogen in the soil decreased. The use of this crop rotation crop structure increased the phosphorus and potassium contents. The simplification of the crop rotations used both in Lithuania and worldwide generally increases the share of cereals in the crop structure. Cereals have a negative impact on soil physical properties, as they increase the spread of monocotyledonous weeds and the incidence of various pathogens, resulting in poor productivity [43,44,45]. Bučienė and Balnytė [46,47] indicate that this effect is especially important in farms that have a narrow specialization, in which the highest-yielding, most profitable crops—cereals—are most often grown. The dominance of one species reduces ecosystem diversity, increases weed incidence, and increases the spread of pathogens. In the study carried out by these authors, barley was grown after as many as six different pre-crops in row crop sequences after maize, sugar beets, and potatoes, as well as being continuously grown after other cereals. In the collection of crop rotations, winter wheat was grown in legume (after the use of a vetch–oat mixture), fallow (after bare fallow), and grass (after the perennial grasses of the first harvest year) sequences. 

In 2016, the amounts of cereals were well matched in all crop rotations, so no significant effect of pre-crops on the yield was found. In 2017, the yields of cereal decreased significantly (by 17.0%) only in the Norfolk crop rotation, in which the pre-crop used for wheat was multiannual leys. In 2016, no significant differences were found, but a 9.8% decrease in the yields was also observed. There may be various reasons for this, such as the increased numbers of weeds or the fact that cultivating perennial grasses for one year is not sufficient to destroy multiannual weeds at the beginning of the vegetation period. The slower mineralization of plant residues may have prevented cereal rooting. In the Norfolk crop rotation, the CO_2_ emissions from the soil were stable throughout the growing season, indicating that plant residues were still intensively degraded by microorganisms.

According to the authors of [48], a stable winter wheat yield, especially when grown in an organic cropping system, can be produced in the sequences of grasses, row crops, and legumes after crops that improve soil fertility. The authors also indicate that the use of a legume sequence is less effective for wheat productivity; however, under our conditions, the highest yield was obtained from sowing wheat after the application of a cattle-manure-fertilized vetch–oat mixture—i.e., in the legume sequence. The author of [47] argues that fertilization with farmyard manure increases cereal productivity for 2 years. The research data also show the positive effect of farmyard manure not only on crop yield but also on the agrochemical, physical, and microbiological properties of soil; soil moisture; and the air regime [48,49,50]. No organic fertilizers were applied in green manure or fodder crop rotations, and in other crop rotations one field was treated with organic fertilizers. Cattle manure was found to influence cereal productivity in different crop rotations; however, fertilization with manure alone is insufficient to ensure a favorable plant nutrition regime. Repšienė et al. [50] point out that the best results can be obtained by combining organic and mineral fertilization. 

## 4. Materials and Methods

### 4.1. Experiment Design and Agricultural Practices

**Figure 6:**
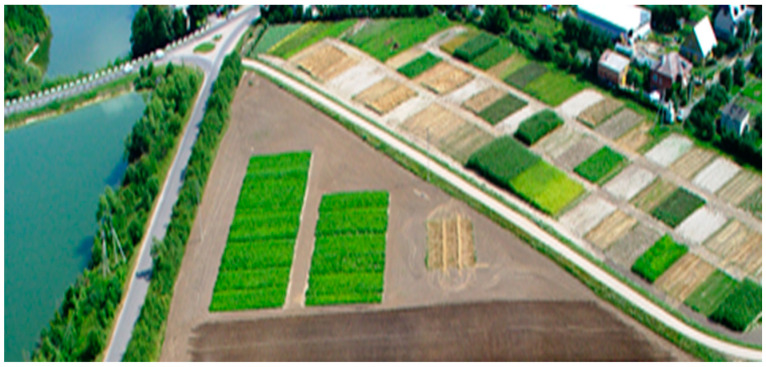
The long-term field experiment conducted at Vytautas Magnus University, Kaunas, Lithuania (54°53′ N, 23°50′ E).

This long-term stationary field experiment (crop rotation collection) was established in 1966 at Vytautas Magnus University Experimental Station in Kaunas, Lithuania (54°53′ N, 23°50′ E), and has been continued until now. This aim of this field experiment was to evaluate the development of crop rotations and their impact on the productivity of agricultural crops, soil properties, and the spread of pests (Figure 6). The long-term impact of crop rotation on the sustainability of agroecosystems was investigated in 9 different crop rotations and in maize and rye monocultures. Continuous bare fallow was used as a control to assess the impact of these different rotations on the physicochemical properties of soil. 

The soil of the experimental site was *Endocalcari-Epihypogleyic Cambisol* (sicco) (CMg-p-w-can). The water regime in the soil was regulated by a closed drainage system and the microrelief leveled. 

In the study, 4 different treatments were applied: 3 crop rotations with different pre-crops and 1 treatment of continuous bare fallow. The cereal crop rotation (CE) pre-crop was a vetch and oat mixture for green forage; the field with row crops (FWR) pre-crop was black fallow; the Norfolk (NOR) crop rotation pre-crop was a clover–timothy mixture; and the control was continuous bare fallow.

All crop rotations were arranged in time and space each year and three replications were applied. Each main plot was 18 m long by 9.60 m wide. The investigation was performed during 2016–2017 on winter wheat (*Triticum aestivum* L.) ‘Skagen’ (200 kg/ha).

Soil samples and measurements were taken from three replications of each crop rotation (cereal (CE), field with raw crops (FWR), and Norfolk (NOR)) and continuous bare fallow (Table 2).

The crop rotations differed not only in their crop sequences but also in their specific input and type of organic matter (shoot, root litter, and cattle manure). In all crop rotations, straw was retained and incorporated into the soil as an organic fertilizer. Undersowing was used as follows: wheat was undersown in the field crop rotation with row crops and in the vetch–oat mixture in the cereal crop rotations. Cattle manure (55 t/ha) was applied to the winter cereal crops in the fields with crop rotations with row crops and the Norfolk crop rotations. Organic fertilizers were incorporated by ploughing at a depth of 15–20 cm. 

Soil tillage in the experiment was carried out according to the common cultivation technologies used for winter wheat. After the first cut, perennial grasses were disked in the field crop rotation with row crops and the Norfolk crop rotation. Ploughing was performed in all crop rotations 10–15 days after the main crop was harvested. All straw was retained and incorporated as organic fertilizer. At the beginning of September, before sowing the soil was cultivated twice, N_8_P_20_K_30_ was applied before the first cultivation. Winter wheat cv. ‘Skagen’ (200 kg/ha) was sown. At the beginning of October, winter wheat was sprayed with the herbicide *Logran 20 WG* at a dose rate of 0.3 l/ha (active ingredient (a.i.) triasulfuron 200 g/kg). At the beginning of the spring vegetation of winter cereals, the plots were fertilized with 200 kg/ha of ammonium nitrate and, after two weeks, additionally fertilized with an amount of 250 kg/ha. The crop stands were sprayed with the growth regulator *Cycocel* 750 SL1 at a level of 1.2 L/ha (a.i. chlormequat chloride 750 g/L) and *Stabilan* at a level of 750 SL (a.i. chlormequat chloride 750 g/L). In the field crop rotation with row crops, a clover–timothy mixture was undersown into winter wheat at the end of March, and the herbicide *MCPA Super* was used at a level of 1.2 L/ha (a.i. MCPA 500 g/L). 

### 4.2. Meteorological Conditions

In the spring of 2016, conditions for the beginning of the vegetative growth of winter cereals were favorable. The hydrothermal coefficient (HTC) in May was 1.09 (optimal precipitation). The HTC in July was 1.62 (excess precipitation) and the air temperature was 1.6 °C higher than the long-term average. In July, there was twice as much rainfall as usual. The monthly HTC was 2.93 (excess precipitation), due to which unfavorable conditions for the normal maturation of cereals and harvesting were created. The air temperature in September 2016 was 1.3 °C higher than the long-term average, while the amount of rainfall was 30.1 mm lower than usual; however, the autumn was cool and very rainy. The air temperature in October was 1.5 °C lower than the long-term average, while the amount of rainfall exceeded the long-term average by 81.9 mm. The amount of precipitation that occurred in November was 20.7 mm more than usual. Significant negative temperatures only occurred in 2017 in January (−3.67 °C), but no severe frosts occurred and there was no snow cover. In winter, the amount of precipitation was close to the long-term average; however, the soil was very wet due to the excess moisture from the autumn. In March, the average air temperature was 4.4 °C higher than the long-term average, but the precipitation was significantly higher, particularly in April, which saw 73.7 mm, 35.3 mm more than usual. Plants suffered from the excess moisture. The HTC in May was 0.29 (very dry), and the temperature was 0.6 °C higher than the long-term average. The HTC in June was 1.78 (excess precipitation). The HTC in July was 1.53 (optimal precipitation), and the temperature was 0.8 °C lower than the long-term average. The summer was favorable for crop vegetation and harvesting. In August, the amount of rainfall was 25.3 mm lower than usual, the temperature was 0.9 °C higher than the long-term average, and the HTC was 1.02 (optimal precipitation).

### 4.3. Methods and Analysis

Soil CO_2_ emissions were measured three times per growing season: 21/06/2016, 19/07/2016, and 26/072016; 16/05/2017, 28/06/2017, and 01/08/2017 Measurements were carried out at the same time every day (from 9.00 a.m. to 4.00 p.m.) and at fixed locations in the field. The soil CO_2_ emissions were measured using an infra-red gas analyzer and the soil surface CO_2_ efflux was also measured (μmol m^−2^ s^−1^) [51,52]. A portable, automated soil gas flux system LI-8100A with an 8100-103 chamber and a LI-8100A analyzer (LI-COR Inc.) was used. In each plot, in spring, rings that were 20 cm in diameter were installed in the soil, from which three measurements were taken for each plot. Soil moisture was measured using the sensor LI-8100-204 (LI-COR Inc.), while soil temperature was measured using the sensor LI-8100-203 (LI-COR Inc.) included in the chamber control kit of the LI-8100A automated soil gas flux system (LI-COR Inc.).

The number of earthworms in the soil was determined after harvesting by a chemical repellent method. In each plot, six frames (50 × 50 cm) were hammered into the soil at a depth of 10 cm. Formalin solution was applied twice every 15 min in 10 L water at a concentration of 0.55%. The earthworms that emerged were collected, counted, and weighed [53]. 

The soil agrochemical properties of the experimental sites were determined before the establishment of the field trial and in each experimental year. By using a soil auger, samples were collected from the plough layer at a depth of 0–25 cm from 15 spots of each plot. Then, the samples were composited (250 g per sample) to provide a representative plot sample for each depth. Organic carbon was measured using a spectrophotometrical method. The quantity of nitrogen per mg kg^−1^ soil was measured using the Kjeldahl method (%), while the contents of phosphorus and potassium were measured using the A–L (Egner–Riehm–Domingo) method. 

The winter wheat yield was measured at the time of harvesting with a Wintersteiger harvester equipped with a weighing and moisture determination system. The grain yield (t ha^−1^) was adjusted to the standard of 14% moisture and 100% purity.

### 4.4. Statistical Analysis

Different crop rotations and continuous bare fallow were statistically compared with each other every investigation year separately. Principal component analysis (PCA based on correlations) was also used to determine the correlation between different parameters depending on the crop rotations used. Principal components analysis was used to create new artificial variables (principal components) based on the variables (features) that we analyzed. Its main advantage is the possibility of visualizing the relationships of individual variables on a two-dimensional graph that shows the coordinate system representing the first two principal components. Based on the position of the vectors in space, the features that are correlated with each other can be determined; the smaller the angle between the vectors, the stronger the positive correlation. When vectors are aligned on the same line but in opposite directions, there is a strong negative correlation between the variables. However, when the vectors are at an angle close to 90° degrees, there is no correlation. Statistical analyses were performed using the Statistica software package Statistica 10 (TIBCO Software Inc., Palo Alto, CA, USA).

The research data were processed through an analysis of variance using the computer program SYSTAT 12 (USA). The Kruskal–Wallis test (*p* < 0.05) was applied to the data that did not correspond to the regularities of normal distribution (e.g., soil CO_2_ emissions). The research data (number of earthworms, their mass, wheat yield) were statistically evaluated using one-way analysis of variance (ANOVA) of the quantitative traits, and the LSD test was also applied. Data on the number and mass of earthworms that did not conform to the normal distribution law were transformed using the Log (X) function before statistical evaluation [54,55].

## 5. Conclusions

1. All the agricultural crops increased the soil CO_2_ emissions compared with the continuous bare fallow field. The highest level of soil CO_2_ emission was found for the Norfolk crop rotations to which perennial grasses and manure were applied. The lowest level of soil CO_2_ emissions was found in continuous bare fallow fields. The pre-crops with a higher organic matter input increased CO_2_ emissions from the soil. The highest contents of organic carbon and total nitrogen were found in the crop rotations where perennial grasses and/or manure were applied: the Norfolk crop rotation and the field with row crops.
2. Significantly higher numbers and masses of earthworms were found in the cereal crop rotation where the pre-crop used was a vetch and oat mixture for green forage. The prevalence of earthworms in these crop rotations was largely influenced by the organic matter input and the content of organic carbon in the soil.
3. Over the study period of two years, we investigated the influence different crop rotations planted in a long-term field experiment (fifty years), revealing the positive effects of perennial grasses and manure fertilization on soil fertility. The highest contents of organic carbon and total nitrogen were found in the crop rotations where perennial grasses and/or manure were applied: the Norfolk crop rotation and the field with row crops. The highest potential for the storage of organic carbon was found in (a) the Norfolk crop rotation, where the crops that reduced soil fertility were rotated with treatments that enhanced soil fertility every year, and (b) the field with row crop rotations, which included perennial grasses and a wide assortment of crops.
4. Winter wheat is more sensitive to the application of different pre-crops; therefore, the influence of crop rotation was significant in all years investigated. The best pre-crop identified for wheat was a vetch and oat mixture fertilized with cattle manure in the short-sequence cereal crop rotation. The productivity of winter wheat increased by 8.8% compared with that of wheat grown in the field crop rotation with row crops in a long sequence.

## Figures and Tables

**Table 1:** The contents of organic carbon, total nitrogen, available phosphorus, and available potassium in soil for wheat crops in 2016 and 2017.

	Pre-Crop (Crop Rotation)	2016	2017
C_org_ (g/kg)	FAL-CONTROL	9.10 ^a^	10.2 ^a^
	LEG-CER (CE)	14.0 ^b^	15.2 ^b^
	FAL-CER (FWR)	13.9 ^b^	14.4 ^b^
	GRS-CER (NOR)	17.3 ^b^	19.5 ^c^
N_sum_ (g/kg)	FAL-CONTROL	0.73 ^a^	0.75 ^b^
	LEG-CER (CE)	1.07 ^b^	1.13 ^a^
	FAL-CER (FWR)	1.13 ^b^	1.05 ^a^
	GRS-CER (NOR)	1.36 ^c^	1.33 ^c^
P_2_O_5_ (mg/kg)	FAL-CONTROL	196.1 ^a^	167.1 ^b^
	LEG-CER (CE)	326.6 ^b^	265.0 ^a^
	FAL-CER (FWR)	298.9 ^b^	225.9 ^b^
	GRS-CER (NOR)	348.2 ^b^	409.5 ^c^
K_2_O (mg/kg)	FAL-CONTROL	210.2 ^a^	248.4 ^a^
	LEG-CER (CE)	453.6 ^b^	667.3 ^b^
	FAL-CER (FWR)	383.6 ^c^	357.2 ^a^
	GRS-CER (NOR)	431.6 ^bc^	580.3 ^b^

Notes: ^a–c^ indicate significant differences between the treatments (*p* ≤ 0.05). FAL-CONTROL, continuous bare fallow; LEG-CER (CE), winter wheat pre-crop was a vetch and oat mixture for green forage while the crop rotation was cereal; FAL-CER (FWR), winter wheat pre-crop was black fallow while the crop rotation was field with row crops; GRS-CER (NOR), winter wheat pre-crop was a clover–timothy mixture while the crop rotation was Norfolk.

**Table 2:** Sequence of crop rotations.

Treatments	Components of Crop Rotation
Continuous Bare Fallow (FAL-CONTROL)	No Crops for 50 Years
Cereal (CE)	(1) **Vetch and oats** (*Vicia sativa* L. + *Avena sativa* L.) **mixture for green forage**, (2) **winter wheat** (*Triticum aestivum* L.), (3) oats (*Avena sativa* L.), (4) spring barley (*Hordeum vulgare* L.).
Field with row crops (FWR)	(1) **Winter wheat** (*Triticum aestivum* L.) + **undersowing**, (2) perennial grasses (*Trifolium pratense* L. + *Phleum pratense* L.) of the 1st harvest year, (3) perennial grasses (*Trifolium pratense* L. + *Phleum pratense* L.) of the 2nd harvest year, (4) winter rye (*Secale cereale* L.), (5) sugar beet (*Beta vulgaris* L.), (6) spring barley (*Hordeum vulgare* L.), (7) oats (*Avena sativa* L.), (8) **black fallow**.
Norfolk (NOR)	(1) **Clover–timothy mixture** (*Trifolium pratense* L. + *Phleum pratense* L.), (2) **winter wheat** (*Triticum aestivum* L.), (3) potatoes (*Solanum tuberosum* L.), (4) spring barley (*Hordeum vulgare* L.).

Note: words in bold indicate the investigated crop (winter wheat) and pre-crop.

## Data Availability

Not applicable.
